# CONFIDENCE IN HEALTH INSURANCE AFFORDABILITY AND DELAYED OR FORGONE HEALTH CARE AMONG ADULTS APPROACHING RETIREMENT

**DOI:** 10.1093/geroni/igz038.041

**Published:** 2019-11-08

**Authors:** Jamie Luster, Renuka Tipirneni, Erica Solway, Preeti Malani, Jeffrey Kullgren, Matthias A Kirch, Dianne Singer, Aaron M Scherer

**Affiliations:** 1 University of Michigan Medical School, Ann Arbor, Michigan, United States; 2 Institute for Healthcare Policy and Innovation, University of Michigan, Ann Arbor, Michigan, United States; 3 Child Health Evaluation and Research Center, University of Michigan, Ann Arbor, Michigan, United States; 4 University of Iowa Carver College of Medicine, Iowa City, Iowa, United States

## Abstract

Recent challenges to the ACA may add uncertainties to decision-making about health insurance. We sought to determine if health insurance affordability concerns were associated with delayed/forgone health care among adults approaching retirement age. In October 2018, the NPHA conducted an online survey of US adults age 50-64. 45% of respondents had little/no confidence in ability to afford health insurance when they retire, and 27% little/no confidence in this over the next year. In the past year, 13% had not gotten medical care and 12% had not filled a prescription because of cost concerns. Controlling for demographic and health characteristics, having little/no confidence in health insurance affordability in retirement/within the next year was associated with delaying/forgoing health care (aOR 2.80, p<0.001). Despite the ACA’s coverage expansions and consumer protections, these findings suggest adults worry about the affordability of health insurance in retirement and may avoid needed health care for that reason.

